# Comparison of efficacy between laparoscopic pectopexy and laparoscopic high uterosacral ligament suspension in the treatment of apical prolapse-short term results

**DOI:** 10.1038/s41598-023-45871-0

**Published:** 2023-10-28

**Authors:** Juan Peng, Shuqing Li, Luwen Wang, Li Yang, Manman Nai, Qingqing Xu, Yuxi Jin, Peng Liu, Lei Li

**Affiliations:** 1 The Department of Obstetrics and Gynecology, The Third Affiliated Hospital of Zhengzhou University, Zhengzhou, China; 2 Zhengzhou Key Laboratory of Endometrial Disease Prevention and Treatment, Zhengzhou, China; 3 The Department of Obstetrics and Gynecology, The First Affiliated Hospital of Zhengzhou University, Zhengzhou, China

**Keywords:** Health care, Medical research

## Abstract

To compare the clinical efficacy of laparoscopic pectopexy and laparoscopic high uterosacral ligament suspension in women suffering from apical prolapse. The clinical data of 170 patients with apical prolapse (POP-Q score ≥ II) treated in the Third Affiliated Hospital of Zhengzhou University from January 2018 to July 2020 were retrospectively analyzed to assess the clinical efficacy of three surgical methods [laparoscopic pectopexy with uterine preservation, laparoscopic pectopexy with hysterectomy, laparoscopic high uterosacral ligament suspension (LHUSLS) with hysterectomy]. Patients were divided into three groups depending on Surgical methods: laparoscopic uterine pectopexy group (n = 23), laparoscopic pectopexy with hysterectomy group (n = 78) and LHUSLS with hysterectomy group (n = 69). The POP-Q points before and after operation were analyzed. The operation-related indices, perioperative periods and post-operative complications were compared. 1. The operation time of laparoscopic uterine pectopexy group was the shortest (p < 0.05). There was no significant difference in the incidence of apical prolapse and new stress urinary incontinence among the three groups during the follow-up period (p > 0.05). 2. The POP-Q points (Aa, Ba, C) in the three groups were better than those before operation (p < 0.05). Laparoscopic pectopexy with hysterectomy group had better Ap, Bp and C points and a longer TVL than LHUSLS with hysterectomy group (p < 0.05). 3. The postoperative PFDI-20, PFIQ-7 and PISQ-12 scores of the three groups were significantly improved than those before operation (p < 0.05). The PISQ-12 scores in laparoscopic uterine pectopexy group were significantly higher than that in the other two groups one year after operation (p < 0.05). The study concludes that laparoscopic pectopexy and LHUSLS can significantly improve the quality of life and sexual function for patients with apical prolapse. One year after operation, laparoscopic pectopexy has a more satisfactory anatomical reduction than LHUSLS with hysterectomy. The laparoscopic uterine pectopexy group had lower postoperative complications and better sexual function than that with hysterectomy group. Laparoscopic pectopexy should be used for the treatment of apical prolapse (POP-Q score ≥ II) patients who aim to better clinical efficacy and sexual function improvement.

## Introduction

Pelvic organ prolapse (POP) is that the normal placement of pelvic organs decline into or out of the vagina, which results from weak muscle and fascial tissue of the pelvic floor. POP is usually accompanied by urination, bowel movements, defecation and sexual dysfunction, thus affecting the quality of life of patients to varying degrees. As global aging, the incidence of POP in women over 50 years old is 30% to 50%^1^. Apical prolapse is a common type of POP including uterine prolapse and vault prolapse. A study has shown that 55% of anterior vaginal wall prolapse cases and 30% of posterior vaginal wall prolapse cases can be corrected simultaneously after apical prolapse reduction^2,3^. The treatment of apical prolapse is quite crucial for POP. Surgery is an important treatment for moderate and severe symptomatic POP when conservative treatment fails.

There have been several surgeries for correcting apical prolapse by laparoscopy, including sacrocolpopexy, sacrospinous ligament fixation, HUSLS and so on. Among these, laparoscopic pectopexy has been applied in clinical practice as a new technique because of its short learning cycle and few operative complications. A number of studies have compared the clinical efficacy of laparoscopic pectopexy with sacrocolpopexy, which shows that its efficacy is more significant^4,5^. Laparoscopic pectopexy requires mesh, while HUSLS depends on autologous tissue. However, there is still a lack of comparative studies between laparoscopic pectopexy and HUSLS. In our study, patients who underwent laparoscopic pectopexy were divided into the uterine preservation group and the hysterectomy group. The clinical efficacy between these two groups and HUSLS were compared. Meanwhile, the pros and cons were further analyzed between mesh and autologous tissue. Laparoscopic HUSLS with uterine preservation wasn’t included in our study because of a small number of cases.

## Materials and methods

We use the pelvic organ prolapse quantification system (POP-Q) to assess the extent of prolapse. The clinical data and follow-up information of 170 patients with apical prolapse (POP-Q score ≥ II) who underwent surgery in the Third Affiliated Hospital of Zhengzhou University from January 2018 to July 2020 was collected by consulting electronic medical records and analyzed retrospectively. Patients were instructed to check by senior attending physicians or gynecologists with senior professional titles in our hospital 3 months, 6 months and 1 year after operation, including POP-Q score, the Pelvic Floor Distress Inventory Short-Form 20 (PFDI-20), the Pelvic Floor Impact Questionnaire-7 (PFIQ-7) and the Pelvic Organ Prolapse/Urinary Incontinence Sexual Function Questionnaire (PISQ-12). With the higher scores of PFDI-20 and PFIQ-7, it means more severe symptoms and a greater impact on life. With the higher scores of PISQ-12, it means better sexual life quality. Among them, 23 patients were treated by laparoscopic pectopexy with uterine preservation (Group A), 78 patients were treated by laparoscopic pectopexy with hysterectomy (Group B), and 69 patients were treated by LHUSLS with hysterectomy group (Group C). Seven patients in Group B and five patients in Group C had undergone total hysterectomy.

The inclusion criteria were as follows: (1) patients (POP-Q score ≥ II) with symptomatic uterine prolapse or vaginal apex prolapse; (2) patients with or without stress urinary incontinence, anterior and posterior vaginal prolapse or cervical prolongation; (3) patients with no obvious surgical contraindications; and (4) patients with surgery performed by several gynecological pelvic floor surgeons with senior titles in our team. The exclusion criteria were as follows: (1) Patients with previous surgical treatment for pelvic floor dysfunction; (2) patients who could not tolerate laparoscopic surgery by preoperative evaluation; (3) patients with acute or chronic inflammation of the reproductive tract, ulcers or urinary tract infections; (4) patients with pelvic organ malignant tumors or other organic lesions; (5) patients with disturbance of blood coagulation; (6) patients with poor postoperative compliance who could not be followed up regularly; (7) patients who were pregnant, lactating and had fertility requirements; and (8) patients with surgical contraindications.

The main surgical methods for treating apical prolapse are sacrospinous ligament fixation, HUSLS, sacrocolpopex, and laparoscopic pectopexy. Informed consent forms were obtained from all of the patients. All the patients were provided counseling on the advantages and disadvantages of surgical techniques, sparing or removing the uterus, and autologous tissue or mesh. Patients choose the surgical method according to their wishes. (1) Regarding whether to retain the uterus, patients were informed that they would not menstruate and had no natural fertility if removing the uterus (for patients who have not yet undergone menopause), and removing the uterus may reduce ovarian function^6,7^. (2) Regarding the placement of mesh or autologous tissue, the hospitalization cost of autologous tissue was relatively low. In contrast, the autologous tissue support function may decline over time. The mesh tissue cost was higher, and there were complications such as mesh exposure, rejection, and erosion^8^. However, the mesh can better restore anatomical structure than autologous tissue^4^, reducing the postoperative recurrence rate^9,10^. Whether to preserve the uterus and place the mesh was made after patients’ consulting with their families.

The physician made a full preoperative evaluation, communicated with the patient and their families, and followed the patient’s decision on whether to remove the uterus and use mesh.

This study was in accordance with the principles outlined in the Declaration of Helsinki. Institutional review board approval: This study was approved by the Ethics Committee of the Third Affiliated Hospital of Zhengzhou University (project identifier: 2021-100-01; approved on December 19, 2021).

### Surgical techniques laparoscopic pectopexy with uterine preservation

**Figure 1:**
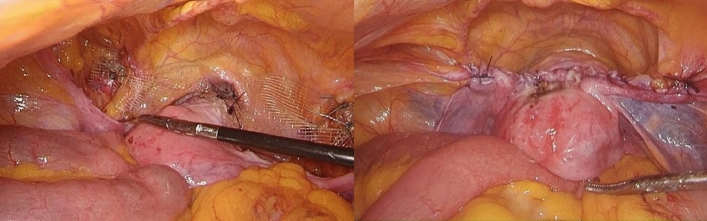
Laparoscopic pectopexy with uterine preservation.

The implantable material used in the abdominal cavity was a PVDF (polyvinylidene fluoride) pelvic floor reconstruction mesh (PV500418F5) produced by FEG Textiltechnik Forschungs-und Entwicklungsgesellschaft mbH in Germany, and the specification is an 18 cm × 4 cm strip mesh. The retrograde peritoneum of the bladder was opened under laparoscopy, the bladder was pushed down, and mesh material was implanted into the abdominal cavity. A Johnson polyester 2-0 delayed absorbable suture was used to suture and fix the center of the mesh to the anterior wall of the cervical canal with an area of 2 cm × 2 cm. The lateral peritoneum was opened along the lateral side of the bilateral round ligament. Then, the loose connective tissue at the medial and inferior sides of the external iliac vein was separated to expose the bilateral iliopectineal ligament, closing the place where the proximal uterine round ligament was brought into the groin. The area of the iliopectineal ligament was 3 cm × 3 cm. The uterus was pushed upward from the vagina to the anterior and posterior walls to restore the normal position, and the tension of the mesh was adjusted to maintain a tension-free state. We sutured both sides of the mesh to the iliopectineal ligament by Johnson Aixikang’s nonabsorbable suture W6977, with 2 stitches on each side. Absorbent sutures were used to suture the pelvic peritoneum to embed the mesh (Fig. 1).

### Laparoscopic pectopexy with hysterectomy

**Figure 2:**
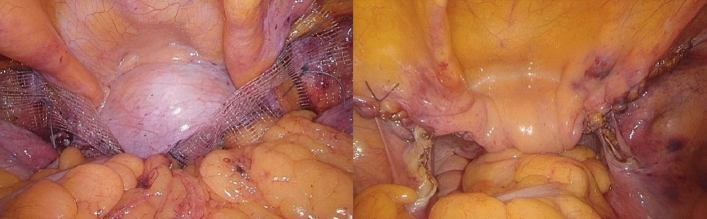
Laparoscopic pectopexy with hysterectomy.

After laparoscopic or transvaginal hysterectomy, Johnson polyester 2-0 delayed absorbable sutures were used to suture and fix the center of the mesh on the anterior and posterior wall of the vaginal stump with an area of 2 cm × 2 cm. The rest of the operation was the same as laparoscopic pectopexy with uterine preservation (Fig. 2).

### LHUSLS with hysterectomy

**Figure 3:**
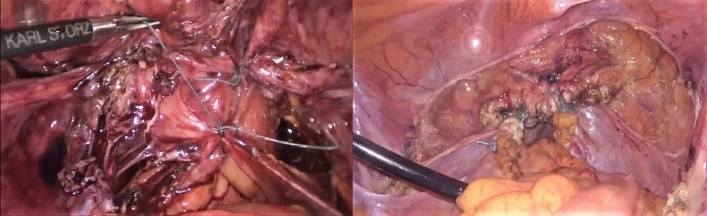
Laparoscopic high uterosacral ligament suspension with hysterectomy.

The vaginal stump was sutured after laparoscopic or transvaginal hysterectomy, and then we identified the course of the ureter and sacral ligament under laparoscopy. The peritoneum was opened between the ureter and sacral ligament, and then the ureter was extrapolated. We opened the rectal space inside the sacral ligament, and the sacral ligament was dissociated upward to 4 cm below the headland of the sacrum. A Johnson Aixikang nonabsorbable suture W6977 was used to continuously suture the middle and lower segments of the sacral ligament to the opposite side of the vaginal tip. Then we tightened the suture and tied a knot. If an obvious rectouterine pouch was found during the operation, we used absorbable sutures to close the pouch (Fig. 3).

From the second day after the operation, all patients began to apply estrogen on the vaginal wall once a day, and the patients were advised to adhere to Kegels exercise.

### Statistical analyses

We used SPSS v.26.0 (IBM Corp., Armonk, NY) for statistical analysis of the study results. The measurement data conforming to a normal distribution are expressed as the mean ± standard deviation ($\overline{{\text{x}}}$ ± s). The differences between the two groups were compared by independent sample t-test, the differences between multiple groups were compared by one-way ANOVA, and multiple pairwise comparisons were performed by LSD-t test. If the data did not conform to a normal distribution, they were expressed as P50 (P25 and P75). The differences between the two groups were compared by the independent sample Z test, the Kruskal–Wallis test was used to compare the differences among groups of independent samples, the Friedman test was used to compare the differences within multiple related samples, and the Bonferroni correction method was used to adjust the significant values after multiple pairwise comparisons. Categorical data are expressed as frequencies (percentages). The differences between the two and three groups were evaluated by the Pearsonχ^2^ test or Fisher’s exact tests. A p < 0.05 was regarded as statistically significant.

## Results

There was no significant difference in age, BMI, parity, menopause, hospitalization days, gynecological operation history or complications among the three groups (p > 0.05). The hospitalization days of LHUSLS with hysterectomy group were significantly longer than the other two groups (p < 0.05). Among these patients, the history of gynecological surgery was mainly tubal ligation, myomectomy, ectopic pregnancy and benign ovarian cyst extirpation. The main complications were hypertension, diabetes and coronary artery disease, and there was no significant difference among the three groups (Table 1). There was no significant difference in the POP-Q stage complicated with cervical prolongation, anterior vaginal wall prolapse, posterior vaginal wall prolapse, old perineal laceration or stress urinary incontinence among the three groups (p > 0.05) (Table 2).

**Table 1:** Comparison of general conditions of the three groups of patients.

	Group A (n = 23)	Group B (n = 78)	Group C (n = 69)	χ^2^/F	p
Age (years)	55.58 ± 4.36	56.64 ± 5.59	56.35 ± 4.87	0.453	0.637
BMI (kg/m^2^)	23.68 ± 2.12	23.63 (22.11, 26.03)	24.41 ± 2.63	3.006	0.222
Parity (n)	2 (2, 2)	2 (1, 3)	2 (1, 3)	1.467	0.48
Menopause n (%)	15 (65.2)	53 (67.9)	47 (68.1)	0.072	0.965
Hospitalization days (days)	10 (9, 11)	10 (10, 12)	11 (10, 13)	11.172	0.004
Gynecological operation history n (%)	5 (21.7)	23 (29.5)	18 (26.1)	0.596	0.742
Complications n (%)	6 (26.1)	26 (33.3)	19 (27.5)	0.78	0.677

**Table 2:** Comparison of special conditions among the three groups.

	Group A (n = 23)	Group B (n = 78)	Group C (n = 69)	χ^2^	p
POP-Q stage n (%)					
Stage II	7 (30.4)	29 (37.2)	29 (42)	1.05	0.592
Stage III	15 (65.2)	43 (55.1)	38 (55.1)	0.828	0.661
Stage IV	1 (4.4)	6 (7.7)	2 (2.9)	1.726	0.412
With cervical extension n (%)	8 (35)	11 (14.1)	12 (17.4)	5.15	0.104
With anterior vaginal wall prolapse n (%)	18 (78.3)	60 (76.9)	49 (71)	0.854	0.652
With posterior vaginal wall prolapse n (%)	7 (30.4)	33 (42.3)	25 (36.2)	1.258	0.533
With old perineal laceration n (%)	4 (17.4)	19 (24.4)	15 (21.7)	0.522	0.77
With stress urinary incontinence n (%)	5 (21.7)	14 (17.9)	14 (20.3)	0.22	0.896

There was no significant difference in combined operation among the three groups (p > 0.05). The operation time of laparoscopic uterine pectopexy group was significantly shorter than that of laparoscopic pectopexy with hysterectomy group, which was considered to be related to uterine preservation, while there was no significant difference between laparoscopic pectopexy with hysterectomy group and LHUSLS with hysterectomy group (p = 0.279). There was no significant difference in the amount of intraoperative bleeding or the duration of postoperative indwelling catheter placement among the three groups (p > 0.05). The incidences of postoperative complications were low and no significant difference among the three groups, including apical prolapse, new stress urinary incontinence, mesh exposure and vaginal stump polyps (p > 0.05) (Table 3). The postoperative pain score of patients was not evaluated in this study.

**Table 3:** Comparison of operation methods, operation-related indices, perioperative complications and short-term postoperative complications among the three groups.

	Group A (n = 23)	Group B (n = 78)	Group C (n = 69)	χ^2^/F	p
Combined operation n (%)					
Anterior vaginal wall repair	12 (52.2)	50 (64.1)	39 (50.7)	1.450	0.484
Posterior vaginal wall repair	8 (34.8)	43 (55.1)	29 (42.0)	4.131	0.127
Old perineal laceration repair	5 (21.7)	19 (24.4)	16 (23.2)	0.075	0.963
TVT-O	3 (13.0)	24 (30.8)	13 (18.8)	4.521	0.104
Operation-related indices					
Operation time (min)	133.57 ± 21.49	146.55 ± 16.88	142.75 ± 25.01	3.385	0.036*
Intraoperative bleeding volume (ml)	50 (20, 50)	50 (30, 50)	50 (20, 90)	3.45	0.178
Duration of postoperative indwelling catheter (days)	2 (2, 2)	2 (2, 3)	2 (2, 2)	2.927	0.231
Perioperative complications n (%)					
Urinary retention	0	0	1 (1.4)	1.472	0.541
Lower extremity venous thrombosis	1 (4.3)	5 (6.4)	3 (4.4)	0.358	0.837
Postoperative fever	1 (4.3)	1 (1.3)	0	2.819	0.253
Postoperative intestinal obstruction	0	0	1	1.472	0.541
Short-term postoperative complications n (%)					
Apical prolapse	1 (4.4)	2 (2.6)	2 (2.9)	1.199	0.840
New stress urinary incontinence	0	1 (1.3)	0	1.186	1.000
Mesh exposure	0	5 (6.4)	–	1.551	0.485
Vaginal stump polyps	–	2 (2.6)	3 (4.3)	1.214	0.695

*There was a significant difference in the operation time among the three groups, including Group A vs. Group B (p = 0.010), Group A vs. Group C (p = 0.073) and Group B vs. Group C (p = 0.279).

The Aa, Ba and C points of the three groups were significantly improved 3 months and 1 year after operation compared with those before operation (p < 0.05). There was no significant difference in the Aa, Ba and C points 3 months and 1 year after operation between laparoscopic uterine pectopexy group and laparoscopic pectopexy with hysterectomy group (p > 0.05). The position of point C decreased in LHUSLS with hysterectomy group 1 year after operation compared with 3 months after operation (p < 0.05), but it did not reach the degree of anatomical apical prolapse recurrence. There was no obvious change in TVL before and after operation among the three groups (Table 4).

**Table 4:** Comparison of the POP-Q indicator points (cm) [M (P25–P75)].

	Before the operation	3 Months after the operation	1 Year after the operation	Before the operation	3 Months after the operation	1 Year after the operation
	Aa			Ba		
Group A	1 (− 2, 2)^b^	− 3 (− 3, − 3)^a^	− 3 (− 3, − 2)^c'^_p1_	2 (0, 3)^b^	− 3 (− 3, − 2)^a^	− 3 (− 3, − 2)^c'^_p1_
Group B	2 (1, 3)^b^	− 3 (− 3, − 2)^a^	− 3 (− 3, − 2)^c'^_p3_	3 (1, 4)^b^	− 3 (− 3, − 2)^a^	− 3 (− 3, − 2)^c'^_p3_
Group C	1(− 0.5, 3)^b^	− 3 (− 3, − 2)^a^	− 3 (− 3, − 2)^c'^_p2_	2 (0, 4)^b^	− 3 (− 3, − 2)^a^	− 2 (− 3, − 2)^c'^_p2_
χ^2^	–	4.613	4.011	–	3.172	5.542
p	–	0.100	0.135	–	0.205	0.063

	Ap			Bp		
Group A	− 3 (− 3, − 3)^b'^	− 3 (− 3, − 3)^a'^	− 3 (− 3, − 3)^c'^_p1_	− 3 (− 3, 0)^b'^	− 3 (− 3, − 2)^a'^	− 3 (− 3, − 2)^c'^_p1_
Group B	− 3 (− 3, − 1)^b'^	− 3(− 3, − 2)^a'^	− 3 (− 3, − 2)^c'^_p3'_	− 3 (− 3, 0)^b^	− 3(− 3, − 2)^a^	− 3 (− 3, − 2)^c'^_p3'_
Group C	− 3 (− 3, 0)^b'^	− 3 (− 3, − 2)^a'^	− 2 (− 3, − 2)^c'^_p2'_	− 2 (− 3, 0)^b'^	− 3 (− 3, − 2)^a^	− 2 (− 3, − 2)^c'^_p2'_
χ^2^	–	4.809	15.062	–	4.283	16.374
p	–	0.090	0.001	–	0.117	< 0.05

	C			TVL		
Group A	2 (1, 3)^b^	− 6 (− 6, − 5)^a^	− 6 (− 6, − 5)^c'^_p1_	8 (8, 8)^b'^	8 (7, 8)^a'^	8 (7, 8)^c'^_p1_
Group B	2 (0, 4)^b^	− 6(− 6, − 5)^a^	− 6 (− 6, − 5)^c'^_p3'_	8 (7, 8)^b'^	8(7.5, 8)^a'^	8(7.5, 8)^c'^_p3'_
Group C	2 (0, 3)^b^	− 6 (− 6, − 5)^a^	− 5 (− 5, − 4)^c^_p2'_	8 (7, 8)^b'^	8 (7, 8)^a'^	8 (7, 8)^c'^_p2_
χ^2^	–	3.419	25.263	–	3.863	9.355
p	–	0.181	< 0.05	–	0.145	< 0.05

Among a group: a: (3 months after the operation) vs. (Before the operation), p < 0.05; aʹ: (3 months after the operation) vs. (Before the operation), p > 0.05; b: (1 year after the operation) vs. (Before the operation), p < 0.05; bʹ: (1 year after the operation) vs. (Before the operation), p > 0.05; c: (1 year after the operation) vs. (3 months after the operation), p < 0.05; cʹ (1 year after the operation) vs. (3 months after the operation), p > 0.05.Among groups: p1: Group A vs. Group B, p > 0.05; p1ʹ: Group A vs. Group B, p < 0.05; p2: Group A vs. Group C, p > 0.05; p2ʹ: Group A vs. Group C, p < 0.05; p3: Group B vs. Group C, p > 0.05; p3ʹ: Group B vs. Group C, p > 0.05.

There was no significant difference in the Ap and Bp points before and after operation in laparoscopic uterine pectopexy group, which was considered to be related to less postoperative posterior vaginal wall prolapse before operation. The Bp points in laparoscopic pectopexy with hysterectomy group were significantly improved 3 months and 1 year after operation compared with those before operation (p < 0.05), but there was no significant difference between 3 months and 1 year after operation (p > 0.05). In LHUSLS with hysterectomy group, the Bp point was significantly improved 3 months after operation compared with that before operation (p < 0.05); the position of the Bp point 1 year after operation was lower than that 3 months after the operation (p > 0.05) (Table 4).

There was no significant difference for every POP-Q indicator point among the three groups 3 months after operation (p > 0.05). The same result for Aa and Ba points between Group A and Group B 1 year after operation (p > 0.05), but the C, Ap, Bp and TVL in laparoscopic pectopexy with hysterectomy group were significantly improved compared with those in LHUSLS with hysterectomy group, suggesting that one year after operation, the anatomical reduction of each point in the laparoscopic pectopexy groups was more satisfactory than those in LHUSLS with hysterectomy group (Table 4).

The PFDI-20 and PFIQ-7 scores of the three groups at 3 months and 1 year after operation were significantly improved when compared with those before operation separately (p < 0.05), but there was no significant difference between 3 months and 1 year after operation (p > 0.05) (Table 5). One year after operation, there was no significant difference in the PFDI-20 and PFIQ-7 scores among the three groups (p > 0.05) (Table 5).

**Table 5:** Comparisons of subjective quality of life and quality of sex life (score).

		Before the operation	3 Months after the operation	1 Year after the operation
PFDI-20	Group A	70 (64, 83)^b^	8.61 ± 1.62^a^	9.48 ± 1.81^c'^_p2_
	Group B	73.68 ± 12.53^b^	9 (8, 10)^a^	9 (8, 10)^c'^_p1_
	Group C	68.52 ± 9.45^b^	10 (8, 12)^a^	10 (9, 11)^c'^_p3ʹ_
	χ^2^	–	–	5.898
	p	–	–	0.052
PFIQ-7	Group A	67.39 ± 13.26^b^	8.78 ± 2.04^a^	9.7 ± 1.55^c'^_p2_
	Group B	69.50 (62.75, 75.25)^b^	9 (7, 10)^a^	9 (8, 10)^c'^_p1_
	Group C	68 (61, 74)^b^	9 (8, 11)^a^	10 (8, 11)^c'^_p3ʹ_
	χ^2^	–	–	3.002
	p	–	–	0.233
PISQ-12	GroupA	22.96 ± 3.937^b^	–	38.87 ± 3.946_p2ʹ_
	Group B	23 (20, 26)^b^	–	33.58 ± 5.166_p1ʹ_
	Group C	22 (20, 25)^b^	–	33.51 ± 3.954_p3ʹ_
	χ^2^	–	–	23.71
	p	–	–	< 0.05

Among a group: a: (3 months after the operation) vs. (Before the operation), p < 0.05; aʹ: p > 0.05; b: (1 year after the operation) vs. (Before the operation), p < 0.05; bʹ: p > 0.05; c: (1 year after the operation) vs. (3 months after the operation), p < 0.05; cʹ: p > 0.05.Among groups 1 year after the operation: p1: Group A vs. Group B, p > 0.05; p1ʹ: Group A vs. Group B, p < 0.05; p2: Group A vs. Group C, p > 0.05; p2ʹ: Group A vs. Group C, p < 0.05; p3: Group B vs. Group C, p > 0.05; p3ʹ: Group B vs. Group C, p < 0.05.

## Discussion

In the United States, 25% of women suffer from at least one pelvic floor disorder^11^. It is generally considered that middle pelvic defects are the most difficult to repair and there is no the best surgical method^12^. At present, the common surgical methods for correcting apical prolapse are sacrospinous ligament fixation, HUSLS and sacrocolpopex^13,14^. Laparoscopic sacrocolpopexy is considered to be the gold standard for the treatment of pelvic floor defects^15,16^. However, the limitations of sacrocolpopexy are gradually exposed in clinical practice, such as difficulties in the operation, long learning curves, possible intraoperative and post-operative complications and dysfunctions. The common complications include defecation disturbance^17^, presacral vascular injury and periostitis^18^. Laparoscopic HUSLS repairs apical prolapse with autogenous tissue. Therefore, there is no risk of mesh-related complications when compared with sacrocolpopexy with mesh, and the vaginal length is not significantly shortened, and the postoperative sexual function of the patients is significantly improved^19^. However, LHUSLS has the risk of ureteral injury or obstruction, S1-4 nerve compression pain or urinary tract infection^20^. The anatomical cure rate of laparoscopic or transvaginal HUSLS is more than 85%^21^. There are abundant blood vessels and nerves around the sacrospinous ligament, and the surgeon must be familiar with the anatomical relationship before operation.

In 2003, Coson et al.^22^ found that the strength of the iliopubic ligament was significantly higher than that of the sacrospinous ligament, so mesh could be sutured and fixed to the iliopubic ligament. In 2011, Banerjee et al.^23^ reported for the first time that laparoscopic iliopubic ligament fixation was used for the treatment of apical prolapse and achieved good clinical effects. Noé et al.^24,25^ conducted a short-term and a long-term comparative study on the clinical effects of laparoscopic pectopexy and sacrocolpopexy in the treatment of apical prolapse. The results showed that the operation time, intraoperative blood loss and defecation disorder of laparoscopic pectopexy were significantly lower than those of sacrocolpopexy. It is a better operation for apical prolapse than sacrocolpopexy^25^.

Noé et al.^26^ conducted an international multicenter clinical study to analyze the security by 501 patients undergoing pectopexy. The results showed that the average operation time was 135 min, the incidence of perioperative complications was 4.2%, and the incidence of severe surgical complications was 1%. In this study, the average operation time of laparoscopic uterine pectopexy group (133.57 ± 21.49 min) was shorter than laparoscopic pectopexy with hysterectomy group (146.55 ± 16.88 min) and LHUSLS with hysterectomy group (142.75 ± 25.01 min). The intraoperative and postoperative complications, the recurrence rate of apical prolapse and the incidence of new stress urinary incontinence among the three groups were all low, indicating that laparoscopic pectopexy and HUSLS had high security and acceptance. Banerjee et al.^23^ and Alkatout et al.^27^ tended to fix the mesh on the cervix. While, there was no significant difference in exposure rate of mesh between laparoscopic uterine pectopexy group and hysterectomy group. Therefore, whether uterine preservation reduced production and exposure of the vaginal apex wound to reduce mesh exposure needs to further study.

The first level in the “three-level theory of pelvic floor” proposed by Delancey is the apex support structure, which is the most crucial support force of the pelvic floor. The uterus and vagina are suspended vertically by the uterine sacral ligament-principal ligament complex^28^. The injury of pelvic floor muscle, fascia, and uterine ligament leads to the decreased supporting tension of pelvic floor tissue, which is a common cause of pelvic organ prolapse. The incidence of POP after hysterectomy is approval 3.2%, which is related to the damage of the apical tissue^29^. The C, Ap, Bp points and TVL of laparoscopic pectopexy with hysterectomy group were better than those of LHUSLS with hysterectomy group 1 year after operation. The clinical efficacy of LHUSLS decreased after one year, and the long-term efficacy may be further reduced, which is not only related to the decrease in suspension strength of the autogenous tissue over time, but also closely related to the apical tissue decreases again due to surgery. More attention should be paid to the fixation of the vaginal apex in patients with hysterectomy. Among the three groups, the C point only in HUSLS with hysterectomy group decreased significantly 1 year compared with 3 months after operation (p < 0.05). This indicates that laparoscopic pectopexy is more satisfactory than LHUSLS with hysterectomy in correcting apical prolapse. Berger et al.^30^ showed that apical prolapse was closely related to anterior and posterior vaginal wall prolapse, and the C point of the patient with anterior and posterior vaginal wall prolapse was lower than that of a normal patient. Whether the decline of point C in our study is related to the decline of Ap and Bp points needs to be further studied. There was no significant difference in the Ap and Bp points in laparoscopic uterine pectopexy group before and after operation, but the Bp point in laparoscopic pectopexy with hysterectomy group was significantly improved compared with it before operation. In general, laparoscopic pectopexy with hysterectomy group had the advantage of improving posterior vaginal wall and apical prolapse. The follow-up time of this study was short and the sample quantity was small. It is worth exploring whether the long-term advantage of pectopexy over HUSLS is more significant.

Tahaoglu et al.^31^ showed that pectopexy can improve the quality of life and sexual function of patients after operation. Our study showed that the PFDI-20, PFIQ-7 and PISQ-12 scores after operation were significantly better than those before operation. The three surgical methods can effectively improve patients’ quality of life in our study. One year after operation, the PISQ-12 score of laparoscopic uterine pectopexy group was significantly better than the other two groups, which is the most ideal for improving patients’ sexual life quality. The PISQ-12 score of patients who underwent laparoscopic pectopexy with hysterectomy was higher than that of patients who underwent LHUSLS, which may be related to the better anatomic reduction of the C point, Ap point and Bp point and longer TVL. One year after operation, although there was no significant difference in the POP-Q index points and TVL between laparoscopic uterine pectopexy group and hysterectomy group, the PISQ-12 score of laparoscopic uterine pectopexy group was higher, suggesting that the sexual quality of life may be related to whether to retain the uterus. Objectively, hysterectomy changes the physiological function of the body, such as damaging the tissue around the top of the vagina, including the uterine-sacral ligament complex and paracervical ring, and it may not be conducive to pelvic structure balance^32^. Subjectively, some patients think that hysterectomy accelerates aging and reduces the characteristics unique to women^33^.

### Limitations

The selected subjects may have data bias because they are limited to a single center. The POP-Q score before or after operation may have difference that of different physicians, and the follow-up time is short, resulting in an imbalance in the number of samples, so it is necessary to conduct a randomized and multicenter long-term follow-up study to observe long-term effects and complications.

## Conclusion

Laparoscopic pectopexy and LHUSLS can effectively improve middle pelvic defects and improve the quality of life and sexual function of patients, but pectopexy is better than LHUSLS. The postoperative complications of patients who underwent laparoscopic pectopexy with uterine preservation were lower, and the sexual function was better than that of patients who underwent laparoscopic pectopexy with hysterectomy.

## Data Availability

The datasets generated during and/or analyzed during the current study are available from the corresponding author upon reasonable request.
